# Signatures of slip in dewetting polymer films

**DOI:** 10.1073/pnas.1820487116

**Published:** 2019-04-19

**Authors:** Dirk Peschka, Sabrina Haefner, Ludovic Marquant, Karin Jacobs, Andreas Münch, Barbara Wagner

**Affiliations:** ^a^Weierstrass Institute for Applied Analysis and Stochastics, 10117 Berlin, Germany;; ^b^Experimental Physics and Center for Biophysics, Saarland University, 66041 Saarbrücken, Germany;; ^c^Mathematical Institute, University of Oxford, Oxford OX2 6GG, United Kingdom

**Keywords:** pattern formation, dewetting, Navier slip, thin films, finite elements

## Abstract

Thin polymer films on hydrophobic substrates are susceptible to rupture and hole formation. This, in turn, initiates a complex dewetting process, which ultimately leads to characteristic droplet patterns. Experimental and theoretical studies suggest that the type of droplet pattern depends on the specific interfacial condition between the polymer and the substrate. Predicting the morphological evolution over long timescales and on the different length scales involved is a major computational challenge. In this study, a highly adaptive numerical scheme is presented, which allows for following the dewetting process deep into the nonlinear regime of the model equations and captures the complex dynamics, including the shedding of droplets. In addition, our numerical results predict the previously unknown shedding of satellite droplets during the destabilization of liquid ridges that form during the late stages of the dewetting process. While the formation of satellite droplets is well known in the context of elongating fluid filaments and jets, we show here that, for dewetting liquid ridges, this property can be dramatically altered by the interfacial condition between polymer and substrate, namely slip. This work shows how dissipative processes can be used to systematically tune the formation of patterns.

## Significance

Dewetting is the hydrodynamic process where a uniform layer of liquid destabilizes and decays into distinct patterns of stationary droplets by virtue of interfacial and intermolecular energies. These patterns can now be predicted theoretically, and their evolution can be followed numerically in striking similarity to experimental results. The droplet arrays observed during the quasistatic evolution are generated by two distinct types of instabilities: droplet shedding of moving liquid rims and satellite droplet pinch-off during the decay of stationary ridges. The mechanism causing the emergence of different droplet patterns is the dissipation at the liquid–solid interface modeled by an effective Navier slip.

The no-slip condition is widely accepted as an appropriate boundary condition for flows of Newtonian liquids sheared along a solid surface. A notable exception arises in the presence of a moving contact line between a viscous liquid and a rigid solid substrate, where the use of the no-slip condition leads to a nonintegrable singularity in the stress field (1, 2). In the past decades, however, it has been shown that thin films of polymer melts can exhibit significant slip when sheared along a substrate, where slip lengths much larger than the film thickness have been observed (3–9).

**Fig. 1.**
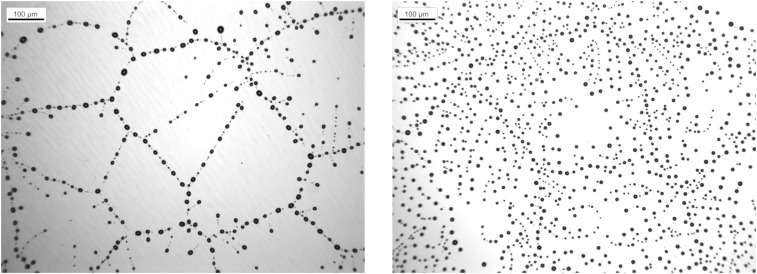
Stationary droplet patterns emerging from a uniform PS(10.3k) film of thickness $h_{\infty }=110\;nm$ after dewetting at $T=120^{○}C$ from a hydrophobically coated Si wafer: (*Left*) AF1600 coating and (*Right*) DTS coating (21).

For retracting rims as they emerge after a hole or trench has opened, the magnitude of slip has a direct impact on the dewetting dynamics. When the slip length is very small or zero, the retraction rate is independent of the size of the growing rim and hence, approximately constant, except for logarithmic corrections (10). For slip that is large compared with the film thickness, viscous dissipation increases with the rim size and gives rise to a $t^{-1/3}$ power law in time t for the retraction (dewetting) rate (11), which has been confirmed experimentally (12–15). In both cases, the moving rim is susceptible to spanwise instabilities (16–18), but for the case where slip is large compared with the film thickness, the dependence of the retraction velocity on the local rim size provides a crucial amplifying mechanism for the instability, which is absent in the no-slip situation (19). As a consequence, the repeated shedding of droplets leaves a characteristic pattern, which is not present for systems with no slip. In either case, the dewetting rims eventually meet to form residual ridges, which in either case, are susceptible to a Rayleigh–Plateau-type instability with a similar dominant wavelength (20). Eventually, this leads to the break up into droplets. As a result of this long-time process, strikingly different droplet patterns are obtained as shown by the experiments depicted in Fig. 1.

The evolution of the polymer melts during these dewetting regimes from early stages after hole formation to the late stages of rupture of the ridges, where the hole boundaries meet, is most appropriately modeled by thin-film models. A systematic asymptotic derivation from full Navier–Stokes equations (22, 23) exploiting the separation between the lateral and normal length scales revealed that the resulting dimension-reduced thin-film model depends on the order of magnitude of slip, leading to two asymptotic distinguished limits: a weak-slip regime and a strong-slip regime.

While thin-film models have been shown to be of great advantage for the analysis of free boundary problems, predicting the evolution over long time and large spatial scales deep into the nonlinear regimes is still a major computational challenge. The primary problem is to resolve the length scales associated with nanoscopic residual layers that remain after the film has dewetted, typically about ∼0.1−1 nm up to thickness of about ∼10−1,000 nm of the growing rim, and to account for the slip length in the range of 1−1,000 nm and the length scale of the resulting instability of $10^{3}-10^{4}\;nm$. By far, the greatest challenge is to make predictions regarding phenomena on the length scales of the instabilities while using numerical solutions with a fine spatial resolution on the length scale of the residual layer.

In this article, we present a numerical algorithm that is able to answer this need featuring a strategy for local adaptivity and an optimized treatment of the intermolecular potential. We will show that the difference in slip lengths indeed leads to the instability patterns seen in experiments. Our numerical solutions also confirm the Rayleigh–Plateau-type instability of the residual ridges during the late phases of the dewetting for both cases, the no-slip and the intermediate-slip cases, with similar wavelengths at the onset, which had been predicted previously based on a linear stability analysis (17, 20). Similar studies were concerned with cases of infinite ridges with (24) and without (25) gravity followed by a broad range of investigations in the literature for this situation using different contact line models and approximations. Numerically, the work by Diez et al. (26) focuses on finite-length ridges but also, includes a review and elucidating comparison of results for the infinite case. Here, most unstable modes are due to varicose perturbations, and the preferred wavelength of the instability is set by the balance of the destabilizing capillary forces, contact line conditions, and for sufficiently viscous liquids, the viscous dissipation. Stability analysis of the effect of slip vs. no-slip on the instability of a stationary ridge shows that both are linearly unstable and have similar wavelengths (17, 20).

Our numerical method allows us to follow the evolution until rupture and predicts significant morphological differences. In particular, it reveals that, for the no-slip case, the breakup is accompanied by the formation of a cascade of satellite droplets that has never been studied before in this context. Moreover, for the intermediate-slip regime, no satellite droplets appear. Interestingly, a closer look at experimental results confirms these predictions. In other contexts, such as liquid jets or fluid filaments, formation of satellite droplets during rupture is well known, and their destabilization was observed to have a rich structure of intermediate asymptotic regimes [e.g., the works by Tjahjadi et al. (27), Eggers and Villermaux (28), and Castrejón-Pita et al. (29)].

We start by introducing the dissipative free surface flow and the relation to pattern formation. Then, we introduce the nonlinear model for dewetting flow and revisit results for dewetting rates and their impact on instabilities in the early stages of the process. We introduce a highly adaptive numerical method that bridges the multiple length scales and timescales and is able to resolve the dewetting with droplet pinch-off. The solutions are discussed, and we compare with experiments.

## Emergence of Dewetting Patterns

Before going into the details of thin-film dewetting, we make some general remarks and simple observations. Assume that the shape of the fluid volume $\Omega \left(t\right)\subset R^{3}$ over a substrate at z=0 and time t is described by a function $h\left(t,x\right)$ such that

$$\Omega \left(t\right)=\left\{\left(x,z\right)\in R^{2}\times R_{+}:0<z<h\left(t,x\right)\right\}.$$ [1]

The wetted substrate area is $\omega \left(t\right)=\left\{x\in R^{2}:h\left(t,x\right)>0\right\}$. Then, the surface energy driving the evolution is defined as

$$E\left(h\right)=\int_{\omega }\left(\gamma_{\ell s}-\gamma_{gs}\right)+\gamma_{\ell g}\sqrt{1+|\nabla h{|}^{2}}\;dx,$$

with $\gamma_{\left\{\ell s,gs,\ell g\right\}}$ being the surface tensions of the liquid–solid, gas–solid, and liquid–gas interfaces, respectively. For incompressible fluids, the evolution of the film height conserves the total volume $V\left(h\right)=\int_{\omega }h\;dx$. For Newtonian fluids with velocity field $u\left(t\right):\Omega \to R^{3}$, this energy decreases along solutions: that is,

$$\frac{d}{dt}E=-D=-\int_{\Omega }2\mu |Du{|}^{2}\;dx\;dz-\int_{\omega }\mu b^{-1}|u{|}_{z=0}^{2}\;dx\leq 0,$$

decomposing the dissipation D into a bulk and substrate part with viscosity μ, Navier slip length b, and symmetric gradient $Du=\frac{1}{2}\left(\nabla u+\nabla u^{⊤}\right)$. Thereby, stationary states are energy minimizers, and a straight-forward minimization of E with constant V shows that minimizers are droplets (i.e., up to translational invariance ω is a disc, and h has constant curvature). Apparently, this assumes that contact lines can slide freely and that no pinning is present (2). However, it is also clear that arrays of droplets as shown in Fig. 1 are not global but local minimizers of E and thereby, also represent viable stationary states. While droplet patterns emerging from the spinodal dewetting feature seemingly random distributions of droplets with details depending on intermolecular interactions (30, 31), droplet distributions in other flow scenarios are reminiscent of the process that leads to their creation (e.g., heterogeneous nucleation, surface instabilities on patterned substrates, droplet production in confined environments, or even dynamically for sliding droplets) (32–38). This raises the natural question: Which mechanism decides what pattern is generated?

This question is even more justified when realizing that the surface energies E leading to both patterns in Fig. 1 are qualitatively identical. Therefore, in the following, we show that physical systems with the same wetting energy but different magnitudes of dissipation (i.e., viscosity and Navier slip) will produce qualitatively different droplet patterns. While the exploration of the nonlinear pattern formation will mainly compare experiments and simulations, the general mechanism responsible for different patterns is the switching of different instabilities due to the dissipation (Movie S1).

## Thin-Film Models and Instability

### Problem Formulation.

After a film has ruptured by nucleation or by external forcing, forming a hole or in a planar-symmetric setting, a trench, the viscous fluid retracts to reduce the overall energy of the liquid–gas, solid–liquid, and solid–gas interfaces. The dewetting process is driven by the intermolecular potential ϕ between the film and the substrate. In the simplest case, it consists of a sum of attractive long-range van der Waals forces and short-range Born repulsion forces, the minimum of which yields the height $h_{⋆}$ that remains in the dry region from which the film of uniform height $h_{\infty }\gg h_{⋆}$ has dewetted. Motivated by Lennard–Jones potentials, one often finds intermolecular potentials (31, 39) of the standard form $\phi \left(h\right)=\bar{\phi }\left(h/h_{⋆}\right)$, where

$$\bar{\phi }\left(h\right)=\frac{S}{n-m}\left(nh^{-m}-mh^{-n}\right),$$ [2]

with m=2,n=8, $\phi \prime \left(h_{⋆}\right)=0$, and $\phi \left(h_{⋆}\right)=S$. For partial wetting, the spreading coefficient $S=\gamma_{gs}-\gamma_{\ell s}-\gamma_{\ell g}$ is negative.

Due to the slow dewetting rates of the polymer films with chain lengths below the entanglement length, the Navier–Stokes equations serve as the underlying model for the viscous fluid with a Navier slip boundary condition,

$$t\cdot \left(2\mu Du\right)n+\mu b^{-1}t\cdot u=0,$$ [3]

for tangential velocity implied by the previous energy dissipation balance. The scale separation of the characteristic height scale H=[z] and typical length scales L=[x] allows for a consistent thin-film approximation using the small parameter

$$\varepsilon =\frac{H}{L},$$ [4]

which leads to the reduction of the Stokes free boundary problem to a problem for the free boundary h(t,x) in closed form. In the following, all quantities are nondimensionalized with this scaling as explained along with details of derivation in ref. 22, which we here only summarize. In particular, we introduce the nondimensional slip length B=b/H.

**Fig. 2.**
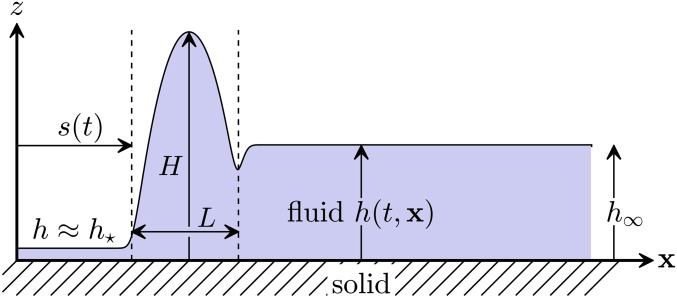
Sketch of solution h(t,x) in the x−z plane.

We seek the scalar function $h:\left[0,T\right]\times R^{2}\to \left[0,\infty \right)$ as in Eq. **1**, where the film height h(t,x) depends on time t∈[0,T] and on space $x=\left(x,y\right)\in R^{2}$ with given initial data $h_{0}\left(x\right)=h\left(t\;=\;0,x\right)$ (Fig. 2). It has been shown that, depending on the magnitude of slip length B, there exist two asymptotic distinguished limits (22) for ε≪1. The first distinguished limit assumes B=O(1) and leads to the weak-slip thin-film model given by the fourth-order parabolic partial differential equation (PDE)

$$\partial_{t}h-\nabla \cdot \left(m\left(h\right)\nabla \pi \right)=0,$$ [5a]

where the mobility is

$$m\left(h\right)=\frac{1}{3}h^{3}+Bh^{2},$$ [5b]

and the generalized pressure π is defined as the functional derivative of the energy functional

$$E\left(h\right)=\int_{R^{2}}\frac{1}{2}|\nabla h{|}^{2}+\phi \left(h\right)\;dx$$ [5c]

with respect to h: that is,

$$\pi =\frac{\delta E}{\delta h}=-\nabla^{2}h+\Pi \left(h\right),$$ [5d]

and $\Pi \left(h\right)=\partial_{h}\phi \left(h\right)$ is the derivative of the nondimensional intermolecular potential. Effectively, by introducing Π, one enforces positivity of $h:\left[0,T\right]\times \bar{\omega }\to \left[0,\infty \right)$ and replaces the complement of the set $\omega \left(t\right)\subset \bar{\omega }\subset R^{2}$ by the region where $h\approx h_{⋆}$ as it is sketched in Fig. 2.

The second distinguished limit assumes that $B=\varepsilon^{-2}\beta$ and leads to the strong-slip thin-film model given by the system of PDEs for the film height $h:\left[0,T\right]\times \bar{\omega }\to \left[0,\infty \right)$ and the lateral velocity $\bar{u}:\left[0,T\right]\times \bar{\omega }\to R^{2}$:

$$\partial_{t}h+\nabla \cdot \left(h\bar{u}\right)\;=\;0,$$ [6a]

$$\text{Re}\left(\partial_{t}\bar{u}+\left(\bar{u}\cdot \nabla \right)\bar{u}\right)\;=\;\frac{1}{h}\nabla \cdot \sigma -\nabla \pi -\beta^{-1}\frac{\bar{u}}{h},$$ [6b]

where Re is the rescaled Reynolds number and the effective shear stress is

$$\sigma =h\left[\nabla \bar{u}+{\left(\nabla \bar{u}\right)}^{⊤}+2\left(\nabla \cdot \bar{u}\right)I\right].$$ [6c]

Two important limiting cases are the no-slip and the intermediate-slip thin-film models. The no-slip model is obtained for B→0 so that the degenerate mobility becomes $m\left(h\right)=\frac{1}{3}h^{3}$. The latter intermediate-slip model is obtained in the limit B→∞ but $\varepsilon^{-2}B\to 0$, which on rescaling of time, produces the mobility $m\left(h\right)=h^{2}$. In this sense, no-slip $m=\frac{1}{3}h^{3}$ mobility and intermediate-slip mobility $m=h^{2}$ are two extremal limits in the slip length B.

It has been shown experimentally and near the linear regime it was also demonstrated theoretically that details of the dynamic and morphological evolution strongly depend on the magnitude of slip at the solid–liquid interface (40). For no slip or weak slip, the liquid accumulates in a growing rim in front of the contact line and destabilizes. The case of strong slip is characterized by very asymmetric retracting rims with a monotone spatial decay toward the unperturbed film for particularly large slip, which undergoes a transition to oscillatory decay as the slip length decreases below a critical value (22, 41). In fact, this observation has been used to identify the strong-slip regime in experiments and also to determine the slip length quantitatively (42–44). The molecular origin of slip in polymer melts was investigated in ref. 45. More details about this topic can be found in the work by Bäumchen and Jacobs (46).

### Dewetting Rates.

We first discuss the dewetting dynamics of a straight rim, where we assume translational invariance $\partial_{y}h\equiv 0$. In the case of no slip, $m\left(h\right)=\frac{1}{3}h^{3}$, the evolution is determined by the region near the contact line where the rim meets the residual film of thickness $h=h_{⋆}$ that remains behind the advancing dewetting front. A careful asymptotic analysis and comparison with long-time numerical solutions (22, 47) reveal that the dewetting rate $\dot{s}\left(t\right)$ is nearly constant. In fact, the position of the contact line s(t) is to leading order given by the dewetting law

$$s\left(t\right)∼\frac{t\;\tan^{3}\left(𝜗\right)}{\ln\left(3\left(h_{\infty }/h_{⋆}\right)t\right)}$$ [7]

as t→∞, where $h_{\infty }=\underset{x\to \infty }{\lim}h$. Physically, this reflects the fact that the size of the rim only has a weak effect on the total friction and hence, in turn, on the dewetting velocity.

For intermediate slip, where $m\left(h\right)=h^{2}$, the evolution of the unperturbed rim is different. At any given size, the rim behaves like a traveling wave but with a wave speed that depends on the width of the rim, which by mass conservation, is proportional to the square root of the distance traveled. A detailed asymptotic analysis (22, 47) that matches the rim to the unperturbed and residual film gives the leading order result

$$s\left(t\right)∼{\left(\frac{9Mb\tan^{5}𝜗}{4h_{\infty }}\right)}^{1/3}t^{2/3}$$ [8]

in the limit t→∞ with M≈0.0272. A similar prediction had been made by Reiter and Khanna (12).

Rims with capillary humps, like the ones that appear here, are known to be subject to Rayleigh–Plateau-like instabilities (20, 24, 26), where the higher capillary pressure in thinner parts squeezes even more liquid into the thicker parts, hence promoting the growth of undulations along the rim. The linear stability of dewetting rims is complicated by the fact that the base state itself grows in time, giving rise to a linearized PDE that cannot be solved exactly using separation of variables. Instead, the linearized PDE can be solved numerically, and the amplification of an initial perturbation can be tracked over time (48). Interestingly, the perturbation evolves into a universal long-time shape that is not sensitive to the initial perturbation. Comparing these shapes reveals an important difference between the no-slip and intermediate-slip cases. The former is much more symmetric and closer to the classical varicose mode observed in the Rayleigh–Plateau instability than the latter (48). Moreover, the maximum amplification is significantly higher for the intermediate-slip case.

These results were analyzed further using an asymptotic sharp interface approach for large rims and a Wentzel–Kramers–Brillouin analysis (also known as WKB analysis, see ref. 49), which established that the long-time dominant wavenumber is given by an equal area rule and is shorter than the prediction by a frozen-mode analysis (50, 51). Furthermore, theory indicates that perturbations of the rim remain small in the no-slip case and for moderate values of B, while in the intermediate-slip case $1\ll B\ll \varepsilon^{-2}$ perturbations grow and develop fingers that eventually pinch off; then, the process repeats itself. The physical explanation for these different manifestations relates to the size dependence of the friction. In the intermediate-slip case, thicker parts of a perturbed rim have a smaller velocity than thinner ones and tend to lag farther behind. This supplies an additional nonlinear enhancement that reinforces the linear Rayleigh–Plateau instability but is essentially absent in the no-slip case, where the dewetting rate is largely independent of the rim size (18, 48, 51). The size dependence also adds another instability mechanism by causing thicker parts of the rim to lag behind thinner parts, thus reinforcing the Rayleigh–Plateau instability and making it more asymmetric (18). A linear stability analysis of the thin-film model predicts the instability to be much more pronounced in the intermediate-slip case than in the no-slip case (48–51).

For slip lengths that are much larger than the film height, the dynamics of the evolution change yet again. In the strong-slip regime, the fluid flow is a plug flow, and the evolution is described by a system of PDEs for the film height and the lateral velocity (6), where the contribution from elongational stresses enters to the same order as the effects from the friction due to slip. Dewetting rim solutions of this model were explored numerically and asymptotically (22, 41). In this regime, the shape of the profile becomes highly asymmetric, with a steep side facing the dewetted area and a much flatter decay to the unperturbed film $h_{\infty }$. This is well reflected in the experimental data for melts with larger polymer chains, where slip is expected to be larger. Interestingly, the change in the balance of stresses leads to an approximately linear dewetting law

$$s\left(t\right)∼\frac{b^{1/2}\tan^{2}\left(𝜗\right)t}{4\sqrt{2}\;h_{\infty }^{1/2}\ln^{1/2}t},$$ [9]

which is another case with an approximately constant velocity of the retracting rim just as in the no-slip case. This suggests that, for very large slip lengths, the rim should become stable again.

## Experimental Setup and Methods

For the experiments, atactic polystyrene (PS; purchased from PSS; molecular weights are as listed in the experiments) is used as a model viscous liquid. The films were produced by spin casting a toluene solution (Selectipur or LiChrosolv; Merck) of PS on freshly cleaved mica sheets. The glassy thin films were then floated onto an ultrapure water (organic impurities of <6 ppb, resistance at $25^{○}C$: <18.2 M Ω cm) surface and were then picked up with hydrophobic Si wafers.

Hydrophobic Si wafers were achieved by two different preparation methods: (*i*) on the cleaned Si surface, a self-assembled monolayer of silane molecules [dodecyltrichlorosilane (DTS); Sigma Aldrich/Merck] was prepared (52), or (*ii*) the cleaned Si wafer was dipped into a solution of a fluoropolymer layer (AF1600; Sigma Aldrich/Merck).

Dewetting is initiated by heating the glassy polymer film above its glass transition temperature. The dewetting of the retracting straight fronts was monitored in situ by optical microscopy on a heating plate (Linkam) or by atomic force microscopy (AFM; Dimension ICON; Bruker).

The dewetted distance was typically obtained from optical micrographs. In AFM experiments, the dewetted distance can also be calculated from 3D scans of the rim on the basis of volume preservation. The values resulting from both approaches were checked for consistency.

Slip lengths have been calculated using the rim profile analysis method (53, 54); structural details, surface roughness values, and wetting properties of the coatings are given in the supplementary material of ref. 40. Polymer film thicknesses have been determined by ellipsometry on the glassy film or by AFM on the edge of a film. The equilibrium contact angles are $𝜗={\left(88\pm 2\right)}^{○}$ and $𝜗={\left(66\pm 2\right)}^{○}$ for AF1600- and DTS-coated substrates, respectively. Comparing typical values for rim height and width, we find typical values in the range ε=0.15−0.3 for the dewetting systems.

**Fig. 3.**
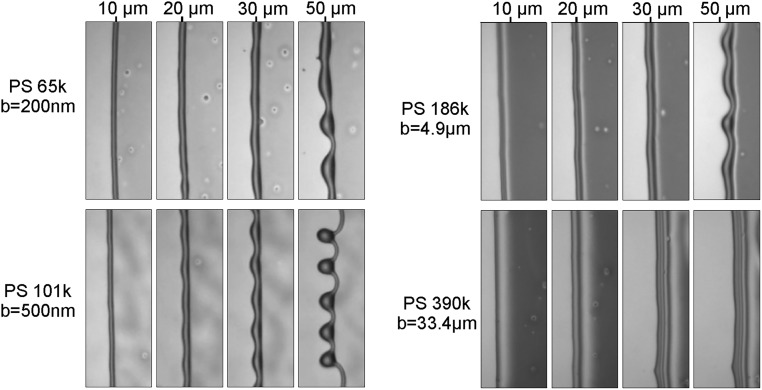
Comparisons of the dewetting behavior of $h_{\infty }=110-nm$-thick PS films for different chain lengths dewetting from AF1600-coated substrate after the rim traveled the distance given above the images. Films are annealed to different temperatures to speed up dewetting in case of films with larger viscosities: PS(65k) at $T=140^{○}C$, PS(101k) at $T=140^{○}C$, PS(186k) at $T=150^{○}C$, and PS(390k) at $T=150^{○}C$. In the chosen systems, the slip length increases with increasing molecular weight, leading to the values indicated next to the corresponding images obtained by fitting the rim profile as reported by Bäumchen et al. (45) or McGraw et al. (55).

In Fig. 3, we show retracting rims with four different chain lengths of the PS film, each thereby corresponding to drastically different slip length b. For the smallest chain length PS(65k), the slip length b is smaller than the rim height H and hence, should correspond to a no-slip situation. As chain length and correspondingly, slip length b increase, we observe a more pronounced instability, as we would expect for a transition from a no-slip scenario to an intermediate-slip scenario. However, if b is increased even further by two orders of magnitudes for PS(390k), the instability is suppressed again. Interestingly, this was predicted for the transition to a strong-slip regime.

For the remaining part of the paper, we will only consider experiments that can be captured with a no-slip (AF1600 coating) or intermediate-slip (DTS coating) regime with PS(13.7k) or PS(10.3k) and use corresponding theoretical models with cubic $m=\frac{1}{3}\;h^{3}$ or quadratic $m=h^{2}$ mobility.

## Numerical Methods

We now explain the intricacies of numerically resolving the multiple length scales of the problem. We focus on the two cases of no slip and intermediate slip, as most of the experimental results regarding the various instabilities fall into either of these two regimes.

Our solution of the thin-film model in Eq. **5** is based on a $P_{2}$ finite element method (FEM), where the fourth-order equation is split into a system of two second-order equations. The FEM uses piecewise quadratic elements and local mesh refinement. We use a semiimplicit time discretization, where only the highest-order derivative is treated implicitly. Therefore, the thin-film equation (Eq. **5**) is multiplied with a test function v and integrated by parts to obtain

$$\int_{\bar{\omega }}\partial_{t}hv+m\left(h\right)\nabla \pi \cdot \nabla v\;dx=0,$$ [10a]

where boundary terms vanish due to the no-flux boundary condition n⋅∇π=0 imposed on $\partial \bar{\omega }$. We also rewrite the pressure π in the weak form as

$$\int_{\bar{\omega }}\pi v\;dx=\int_{\bar{\omega }}\nabla h\cdot \nabla v+\Pi \left(h\right)v\;dx,$$ [10b]

where again, we used integration by parts and n⋅∇h=0 on the boundary $\partial \bar{\omega }$. In particular, this statement of the PDE implies conservation of volume

$$\frac{d}{dt}\int_{\bar{\omega }}h\left(t,x\right)\;dx=0,$$

which can be seen when selecting v=1 in the continuous or discrete weak formulation (Eq. **10a**). Evaluating the solution at discrete times $h_{n}\left(x\right)=h\left(n\tau ,x\right)$, we use the time discretization $\partial_{t}h=\tau^{-1}\left(h_{n}-h_{n-1}\right)$. This allows us to rewrite the weak form of the thin-film model in Eq. **10** so that we seek $\left(h_{n},\pi \right)\in W$ such that

$$\int_{\bar{\omega }}h_{n}v+\tau \;m^{*}\;\nabla \pi \cdot \nabla v\;dx=\int_{\bar{\omega }}h_{n-1}v\;dx,$$ [11a]

$$\int_{\bar{\omega }}\pi w-\nabla h_{n}\cdot \nabla w\;dx=\int_{\bar{\omega }}\Pi^{*}w\;dx,$$ [11b]

which needs to hold for all (v,w) from a suitable function space W and with initial data $h_{0}$. When we define the mobility $m^{*}=m\left(h_{n-1}\right)$ and $\Pi^{*}=\Pi \left(h_{n-1}\right)$, this becomes a semiimplicit time discretization.

**Fig. 4.**
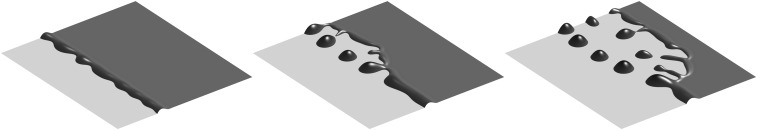
Simulated pattern formation with intermediate-slip $m=h^{2}$: 3D view with light areas showing dry regions, whereas elevated dark areas show the liquid–air interface.

**Fig. 5.**
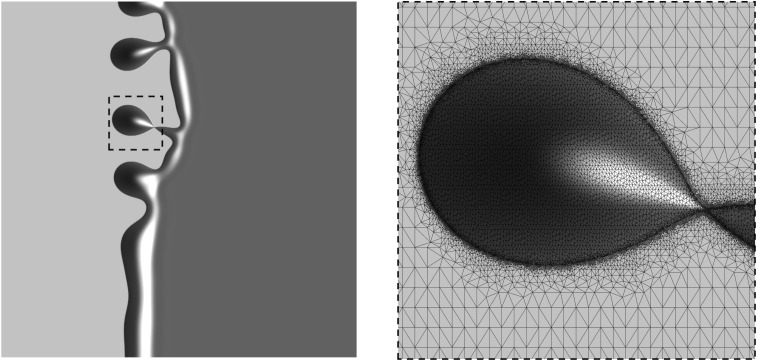
(*Left*) Dewetting rim during pinch-off of a single droplet highlighted with dashed lines and (*Right*) magnification of this droplet and corresponding locally refined triangulation. The mesh consists of 92, 272 vertices, which require us to solve for 2×368, 423=736, 846 unknowns $\left(h_{n},\pi \right)$ for the $P_{2}$ FEM discretization at each time step.

The FEM constitutes a method for the construction of a finite-dimensional subspace $W_{h}\subset W$, where we have an admissible decomposition $\bar{\omega }={⋃}_{k=1}^{N}T_{k}$ of the domain into triangles $T_{k}$, on which we define $W_{h}$ as the space of continuous functions that are piecewise quadratic on each triangle (i.e., $P_{2}$ finite elements). Then, we seek a discrete solution $\left(h_{n},\pi \right)\in W_{h}$ of Eq. **11** valid for all $\left(v,w\right)\in W_{h}$. The integrals appearing in Eq. **11** are solved exactly or using a seven-point Gauss quadrature. A typical droplet pattern emerging from the simulation with $m=h^{2}$ is shown in Figs. 4 and 5.

The precursor thickness $h_{⋆}$ in the potential of Eq. **2** needs to be chosen much smaller than any droplet size we intend to resolve. Then, the solution h will feature large regions, where the solution is either almost constant $h\approx h_{⋆}$ or the solution is smooth and $h\gg h_{⋆}$ as shown in Fig. 2. However, where those regions meet, the solution features a kink, which should be resolved in the triangulation (compare with Fig. 5). We perform a heuristic local mesh refinement in these connecting regions where the contact line is situated. When constructing a new mesh, we start from a coarse base mesh and determine which of its elements are crossed by the contact line of the previous solution. Those elements are refined by inserting additional vertices to an extent that the kink is resolved again. Furthermore, we make sure that neighboring elements are also refined, so that the contact line remains in the refined region of the mesh for a number of time steps. Based on the set of newly created vertices, we perform a new triangulation and interpolate the old solution onto the new triangulation.

**Fig. 6.**
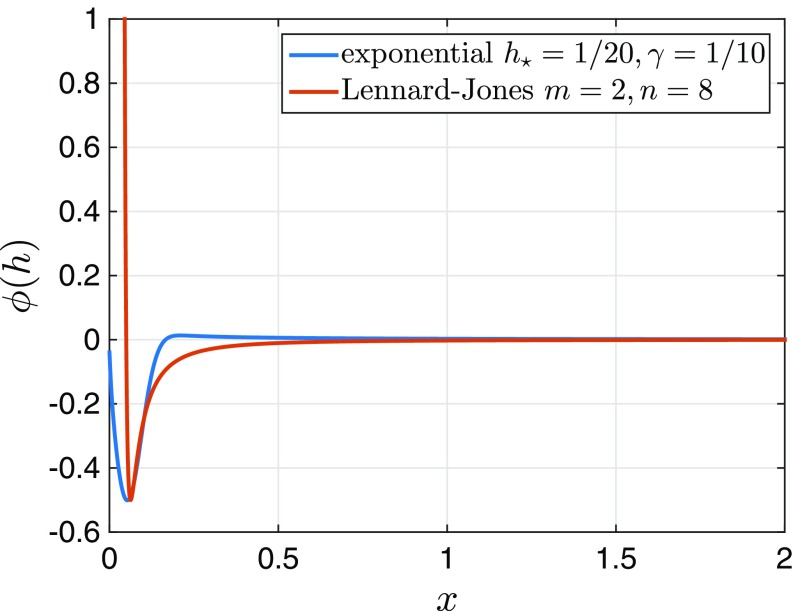
Lennard–Jones potential Eq. **2** compared with the much shorter-ranged exponential potential Eq. **12**, both with $h_{⋆}=1/20$ and $\varepsilon_{⋆}=1/16$. Note that, in the numerics we rescaled so that at the contact line the slope is |∇h|=1, leading to a nondimensional potential with $\phi \left(h_{⋆}\right)=-1/2$.

To allow the control of the minimum and the derivatives of ϕ separately via $h_{⋆}$ and $\varepsilon_{⋆}$, it is advantageous to work with an alternative potential representation of the form $\phi \left(h\right)=\hat{\phi }\left(\left(h-h_{⋆}\right)/\varepsilon_{⋆}\right)$ with

$$\hat{\phi }\left(s\right)=\frac{1}{2}\left(\frac{\gamma }{1+s}-\left(1+\gamma \right)\exp\left(-s^{2}\right)\right),$$ [12]

which has similar properties as the rescaled version of Eq. **2**. In particular, with this potential, we still have the same Γ convergence property as $h_{⋆},\epsilon_{⋆}\to 0$. This abstract statement ensures that equilibrium contact angles are maintained and that the energetic contributions of ϕ from Eq. **12** and Eq. **2** coincide in this limit. We note that, for 0<γ≪1, the minimum slightly shifts away from $h_{⋆}$; however, one gains a slightly stabilizing potential with *ϕ*″ > 0 for $h\gg h_{⋆}$. In addition, we point out that we monitor that the minimum of the solution $\min_{x}h\left(t,x\right)$ never violates the nonnegativity requirement for the given choice of ϕ(h) and parameters $h_{⋆},\varepsilon_{⋆}$ (Fig. 6), in particular, by keeping the time step size τ sufficiently small. We also refer to previous mathematical studies (56, 57) that incorporate the nonnegativity property into their numerical scheme.

## Initial Data for Rims

We still need to specify the initial data $h_{0}\left(x\right)$ for the numerical simulation to describe the various stages of the dewetting process. In experiments, the dewetting is initiated from a uniform flat layer bounded by a nearly straight edge. When the sample is heated, the layer liquefies, and the edge becomes a moving contact line of the dewetting process. In simulations, we choose the supporting domain sufficiently large $\bar{\omega }=\left[0,500\right]\times \left[0,600\right]$; using $h_{⋆}=1/20$, we represent the uniform layer with the nearly straight contact line at $x_{0}$ with initial height

$$h_{0}\left(x\right)=h_{⋆}+\frac{1}{2}\left(h_{\infty }-h_{⋆}\right)\left(1+\tanh\left[\frac{x-x_{0}\left(y\right)}{\Delta }\right]\right),$$ [13]

and the smooth initial contact line position is represented by $x_{0}\left(y\right)=20+\sum_{n=1}^{50}a_{n}\cos\left(n\pi y/600\right)$. With Δ=1/2, one can interpret $h_{0}$ to be an approximation of a step-like profile, where slight corrugations of $x_{0}$ along the y direction are introduced using the normally distributed coefficients $a_{n}$ with zero mean and SD 1/10. This choice is then maintained throughout all dewetting simulations for both mobilities. Due to the introduced scaling, we have $h_{\infty }=1$.

**Fig. 7.**
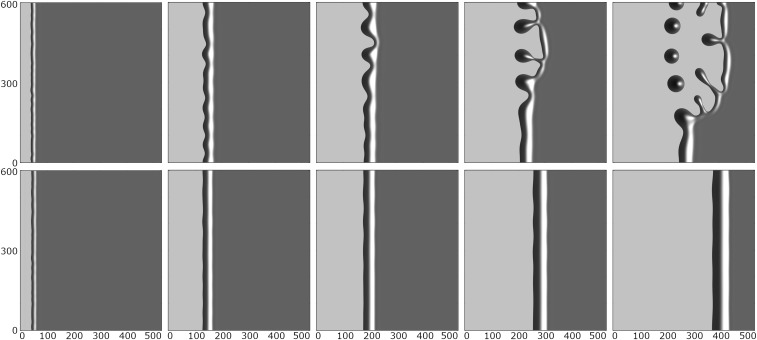
Numerical solutions on $\bar{\omega }=\left[0,500\right]\times \left[0,600\right]$ ($h_{⋆}=1/20$, γ=1/10) for (*Upper*) $m=h^{2}$ (intermediate-slip B→∞) and (*Lower*) $m=\frac{1}{3}h^{3}$ (no-slip B=0) for initially slightly perturbed straight front and time progressing from left to right shown for similar rim progression (Movie S1).

**Fig. 8.**
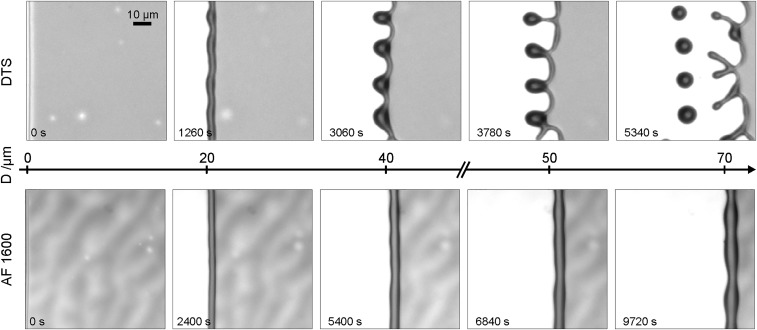
Series of micrographs from experiments with thin films (*Upper*) dewetting from a hydrophobized Si wafer covered with a silane monolayer DTS with b=1 μm (intermediate slip) and (*Lower*) dewetting from an AF1600-covered Si wafer with b=40 nm (no slip). In both series, a $h_{\infty }=100\;nm$ thin PS(13.7k) film dewets at $T=120^{○}C$. In both series, undulations are formed along the rim; however, only in the intermediate-slip case above, budding is observed that later leads to fingers and a pinch-off of droplets. Comparing rims that have traveled a similar distance ensures that only rims of similar volumes are evaluated. Reprinted with permission from ref. 19. Copyright 2015 by the American Physical Society.

To study the systematic effect of the slip boundary condition on dewetting patterns, we study the contact line instability of moving rims and stationary ridges for the mobility functions $m\left(h\right)=\frac{1}{3}h^{3}$ (no slip) and $m\left(h\right)=h^{2}$ (intermediate slip). In Fig. 7, the evolution of the rims for no slip and intermediate slip is shown at times where they have accumulated a similar volume, starting with the same initial film thickness $h_{\infty }$ as given by the experiments. This corresponds to the experimental results in Fig. 8, where the evolution of the rims is shown at the same distance measured from the initial coordinates. The distinctive dewetting patterns for the intermediate-slip case are observed, while for the no-slip case, only shallow oscillations will occur in excellent comparison with the experimental results in Fig. 8.

## Breakup of Liquid Ridges

Toward the final stages of dewetting process, for long times and domain size far beyond $\bar{\omega }$, the rims approach each other and merge, yielding a polygonal network of ridges. As is known from previous studies, stationary ridges are susceptible to a Rayleigh–Plateau instability for both no-slip and intermediate-slip boundary conditions at the substrate. Both the no-slip case and the intermediate-slip case have been extensively investigated in the literature (20, 24, 26).

**Fig. 9.**
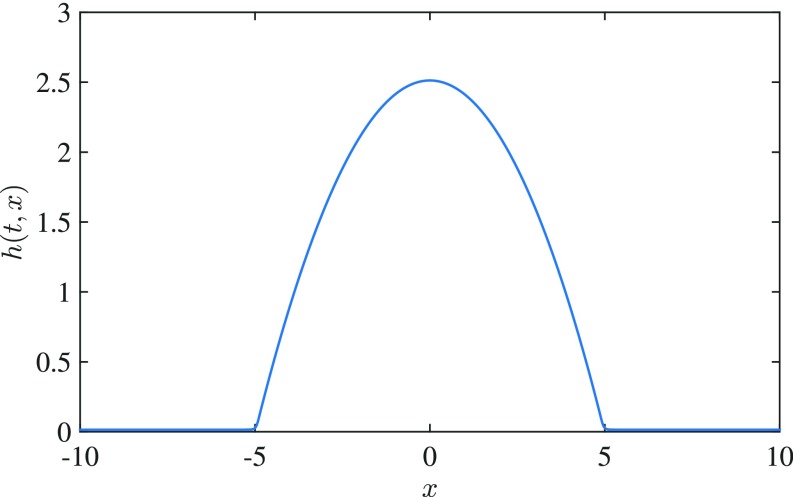
The 1D steady-state $h_{stat}$ with $\varepsilon_{⋆}=1/20$ and $h_{⋆}=1/80$.

To study the instability of a capillary ridge, we first compute a 1D stationary solution $h_{stat}\left(x\right)$ (i.e., a time-independent 2D solution of Eq. **5** with $\partial_{t}h\equiv 0$, $\partial_{y}h\equiv 0$, and constant thickness $h\to h_{⋆}$ as x→±∞ as shown in Fig. 9, with a symmetric and approximately parabolic shape around the maximum). Then, we study the linear stability with respect to perturbations

$$h\left(t,x\right)=h_{stat}\left(x\right)+\delta h_{1}\left(x;k\right)\exp\left(iky+\sigma t\right)$$ [14]

with fixed wavenumber k, which returns an eigenproblem for the perturbation $h_{1}$ of the base state (20, 51). We select the most unstable mode $k_{\text{max}}$ and define as initial data

$$h_{0}\left(x\right)=h_{stat}\left(x\right)+\delta h_{1}\left(x;k\right)\cos\left(ky\right)$$ [15]

with a sufficiently small δ so that the nonnegativity of $h_{0}$ is also not violated. In *SI Appendix* , *SI Text* and Figs. S1–S3, we present the details of the linear stability analysis and show that solutions of the nonlinear problem follow the predicted exponential amplification while the linear regime is valid. Furthermore, we checked that the alternative choice of potential Eq. **12** compared with the standard choice Eq. **2** has no visible impact on the linear stability as long as the minimal value $\phi \left(h_{⋆}\right)$ and the minimal height $h_{⋆}$ are the same.

However, we are interested in using the numerical simulation as a tool to observe features in the nonlinear regime that are distinctive features of the mobility law. Therefore, to resolve finer features of the ridge shape (e.g., secondary or tertiary droplets), the general form of the intermolecular potential is the same, but we use a smaller $h_{⋆}=1/80$.

We now consider nonlinear simulations using the stationary ridge with very small monochromatic perturbations as initial data. Specifically, we consider an unstable perturbation with $k_{\text{max}}$ and carry out simulations with the mobilities $h^{2}$, $\frac{1}{3}h^{3}$.

As long as the perturbations are small enough, the evolution of the corrugations in the nonlinear model closely follows the predictions from the linear stability analysis. After the perturbations become comparable with the size of the rim, nonlinear terms become relevant, and the growth rate changes.

**Fig. 10.**
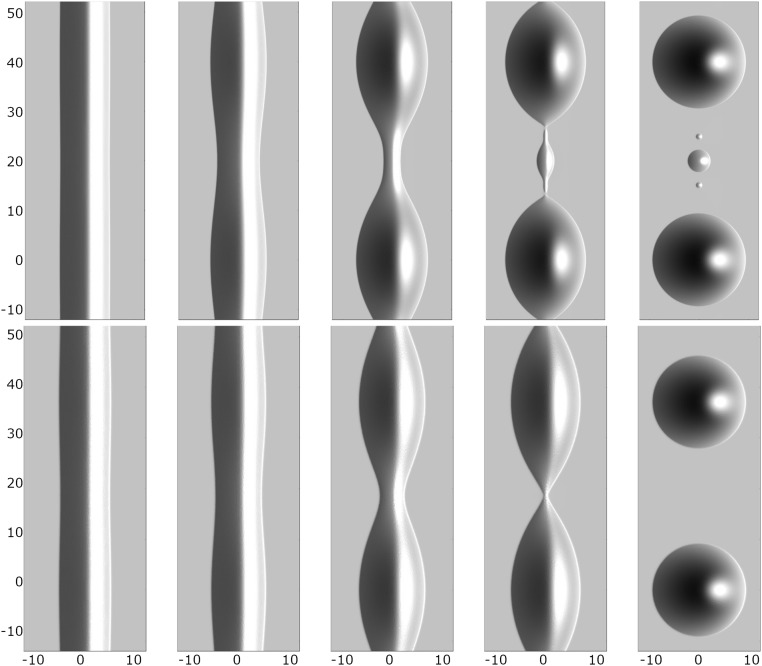
Numerical solutions showing ridges with (*Upper*) no slip $m\left(h\right)=\frac{1}{3}h^{3}$ and (*Lower*) intermediate slip $m\left(h\right)=h^{2}$. The initial data are $h_{0}\left(x\right)=h_{stat}\left(x\right)+\delta h_{1}\left(k_{\text{max}};x\right)\cos\left(ky\right)$, with time increasing from left to right. The wavenumber $k_{\text{max}}$ is the one with the largest amplification from Eq. **14**, and the domain $\bar{\omega }=\left[-12,0\right]\times \left[0,\pi /k_{\text{max}}\right]$ is then extended to $\left[-12,12\right]\times \left[-\pi /k_{\text{max}},3\pi /k_{\text{max}}\right]$ using the implied symmetry of the solution (Movie S1).

**Fig. 11.**
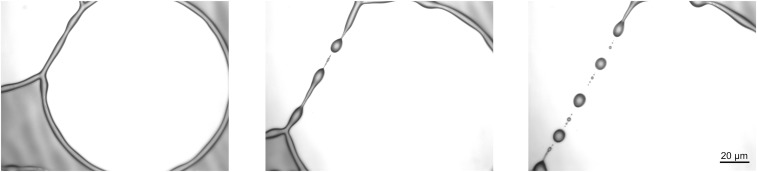
Experimental results. Close-up view of the late-stage ridge of PS(10.3k) on AF1600 with time progressing from left to right showing decay into satellite and subsatellite droplets. The initial film height is $h_{\infty }=115\;nm$, and b=40 nm. With B=b/H and H corresponding to rim height, this gives rise to the no-slip regime (21).

Regarding the morphological evolution of the ridges for the no-slip and intermediate-slip cases, our numerical simulations show qualitatively and quantitatively very similar behavior during the linear regime. However, deep into the nonlinear regime, the breakup into droplets follows different scenarios. In the no-slip case, a cascade of satellite droplets emerges, while for the intermediate-slip case, they disappear. Starting with the same initial condition, the evolution for both cases is shown in Fig. 10. Interestingly, this different behavior is also observed in our experimental results as seen in Fig. 11. The instability mechanisms causing these different patterns are encoded in the behavior near the self-similar pinch-off (e.g., refs. 58 and 59 are related theoretical studies of thread breakup).

## Conclusion and Discussion

We have introduced a highly adaptive finite element-based numerical approach that correctly captures the complex dewetting process described by a class of thin-film models with degenerate mobilities. We showed that, for the no-slip condition, the droplet pinch-off is absent during the retraction of the rim, while for the intermediate-slip case, self-repeating droplet pinch-off occurs in excellent agreement with experimental observations. The ability to resolve the different length scales for long timescales also enables the prediction of phenomena, such as the formation of satellite droplets, as a function of the mobility. The emergence of satellite droplets is well known during the break up of liquid jets and the related problem of liquid filaments. For the latter problem, destabilization is due to the difference in the axial contribution to the capillary pressure between thicker and thinner parts. In this system, the pressure is higher in the thinner parts and squeezes the liquid into the bulges, thus increasing the perturbation until the filament breaks up. Apart from the huge literature on experimental studies, the problem has sparked numerous numerical and analytical investigations (28, 60, 61). In particular, the work by Tjahjadi et al. (27), where the emergence of satellite droplets was captured numerically, and the highly the accurate numerical schemes developed by Kim et al. (62) improved the understanding the underlying physical processes considerably.

For the situation of a ridge that destabilizes on a solid substrate, the additional influence of the substrate enters the linear stability analysis, in particular through the contact angle dynamics. It will be interesting to investigate analytically the rupture behavior for this problem to help understand the influence of the boundary condition at the substrate. Similarly, it remains an interesting open question how the dissipation due to a dynamic contact angle would affect the whole pattern formation process. Such a study would certainly require even more sophisticated finite element techniques (63, 64).

The decision about the pathway leading to specific droplet patterns is then often decided by the specific influence that the dissipation has on flow instabilities. In this study, these instabilities are the shedding of droplets from moving rims and the symmetry of the Rayleigh–Plateau instability leading to satellite droplets, and they are influenced by the magnitude of the interface dissipation encoded in the Navier slip condition.

We conclude that, by controlling dissipative effects in this dewetting films system, we can steer pattern formation without changing the driving forces.

## Supplementary Material

Supplementary File

Supplementary File
