# Supplementary material for: Signatures of slip in dewetting polymer films
Source: Proc Natl Acad Sci U S A. 2019 Apr 19;116(19):9275–84. doi: 10.1073/pnas.1820487116 (PMC6510987; doi:10.1073/pnas.1820487116)
Supplement: Supplementary File [file pnas.1820487116.sapp.pdf]

PNAS
www.pnas.org

Supplementary Information for

Signatures of slip in dewetting polymer films

Dirk Peschka, Sabrina Haefner, Ludovic Marquant, Karin Jacobs, Andreas Münch, Barbara Wagner

Dirk Peschka.
E-mail: peschka@wias-berlin.de

This PDF file includes:

Supplementary text
Figs. S1 to S3
Caption for Movie S1
References for SI reference citations

Other supplementary materials for this manuscript include the following:

Movie S1

Supporting Information Text

Linear stability for ridges

In the following we provide some details for the linear stability analysis of 1D liquid ridges with respect to 2D varicose perturbations and show the impact of using different mobilities and potentials. Consider the following fourth-order parabolic partial differential equation for the height $h(t, \mathbf{x})$

$$\partial_t h - \nabla \cdot (m(h)\nabla\pi) = 0, \qquad \pi = -\nabla^2 h + \Pi(h), \tag{1}$$

depending on time t and space $\mathbf{x} = (x, y) \in \mathbb{R}^2$ for given initial data $h_0(\mathbf{x}) = h(t = 0, \mathbf{x})$. With $\Pi(h) = \phi'(h)$ we denote the derivative of an intermolecular potential $\phi(h)$ and a degenerate mobility $m(h)$, *i.e.*, $m(h) = h^3$ (no-slip) and $m(h) = h^2$ (intermediate-slip) with $m \to 0$ as $h \to 0$. Often, one finds standard potentials of the form $\phi(h) = \bar{\phi}\,(h/h_\star)$ with

$$\bar{\phi}(h) = \frac{1}{2}(n-m)^{-1}\big(nh^{-m} - mh^{-n}\big), \tag{2a}$$

where $n = 2$, $m = 8$. Alternatively, we consider $\phi(h) = \widehat{\phi}\,((h - h_\star)/\varepsilon_\star)$ with

$$\widehat{\phi}(h) = \frac{1}{2}\left(\frac{\gamma}{1+h} - (1+\gamma)\exp(-h^2)\right). \tag{2b}$$

This has the advantage that the minimum of ϕ and the decay $\phi \to 0$ as $h \to \infty$ can be controlled separately via $h_\star$ and $\varepsilon_\star$, respectively. Stationary ridges appear as time-independent solutions $h(t, \mathbf{x}) = h_{\mathrm{stat}}(x)$ of Eq. (1), where $h\big(t, (x, y)\big) \to h_\star$ as $x \to \pm\infty$. Such solutions are invariant with respect to translations, thereby defining a family of solutions using the location x_0 and height of the maximum $h_{\mathrm{stat}}(x_0)$ (corresponding to ridge volume). An example for $h_{\mathrm{stat}}(x)$ is provided in the manuscript. We now consider perturbations of $h(t, \mathbf{x}) = h_{\mathrm{stat}}(x)$, where for $|\delta| \ll 1$ we assume

$$h(t, \mathbf{x}) = h_{\mathrm{stat}}(x) + \delta\, h_1(x; k) \exp\big(iky + \sigma(k)t\big), \tag{3}$$

which leads to the eigenvalue problem $L_k u_k = \sigma(k) u_k$ with $u_k = h_1(\cdot; k)$, where L_k is the linear fourth-order elliptic operator

$$L_k u_k = -\partial_x[m(h_{\mathrm{stat}})\partial_x(\partial_{xx}u_k - k^2 u_k - \phi''(h_{\mathrm{stat}})u_k)] + k^2 m(h_{\mathrm{stat}})[\partial_{xx}u_k - k^2 u_k - \phi''(h_{\mathrm{stat}})u_k]. \tag{4}$$

and $u_k(x) \to 0$ for $x \to \pm\infty$, cf. (1). Note that L_k depends on the stationary solution h_{stat}. The resulting operator is discretized using finite-differences on a uniform mesh and by splitting the problem into a system of two second-order equations A_k. This problem is solved using a standard solver for generalized eigenvalue problems $A_k(u_k, w_k)^\top = \sigma(k) B_k(u_k, w_k)^\top$. In Fig. S1 the dispersion relation $\sigma(k)$ is shown for no-slip and intermediate-slip mobility. The corresponding eigenfunctions are displayed Fig. S2. The dispersion relation and eigenmodes are not visibly affected by the alternative choice of potential Eq. (2b) compared to the standard choice Eq. (2a), as long as the minimal value $\phi(h_\star) = -1/2$ and the minimal height $h_\star$ are retained. The amplification $\sigma(k)$ is compared to the nonlinear behavior in Fig. S3. In all shown cases the base state h_{stat} has the same position x_0 and height $h_{\mathrm{stat}}(x_0)$.

References

1. King J, Münch A, Wagner B (2006) Linear stability of a ridge. *Nonlinearity* 19:2813–2831.

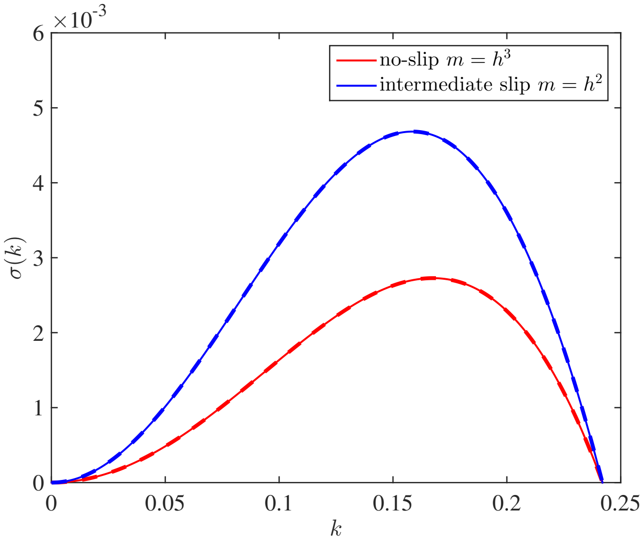

Fig. S1. Dispersion relation $\sigma(k)$ for linear stability analysis of a ridge for no-slip $m = h^3$ (red) and intermediate-slip $m = h^2$ (blue) mobility. Full lines for alternative potential $\hat{\phi}$ and dashed lines for standard potential $\bar{\phi}$ overlap and thereby show that they give the same results in the linear regime. Solving the eigenvalue problem $\mathrm{Eq.}\ (4)$ that results from $\mathrm{Eq.}\ (3)$ for $\sigma(k)$ numerically reveals that there is a range $0 < k < k_c$, where for no-slip and intermediate-slip mobility one positive real eigenvalue σ exists. That eigenvalue corresponds to varicose perturbations of the ridge, as can be seen from the corresponding eigenfunction u_k in Fig. S2. Note that the most preferred wavenumber $k_{\text{max}} = \arg\max_k \sigma(k) = (0.168, 0.159)$ only differs slightly between the no-slip and intermediate-slip case, respectively. The corresponding maximal amplifications $\sigma_{\text{max}} = \max_k \sigma(k) = (2.7 \cdot 10^{-3}, 4.7 \cdot 10^{-3})$ are consistent with the nonlinear evolution shown in Fig. S3 for no-slip and intermediate-slip mobility, respectively.

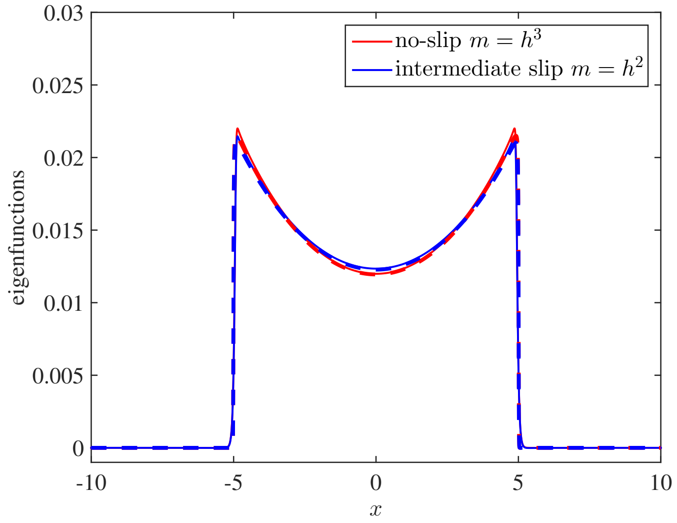

Fig. S2. Normalized eigenfunctions $u_{k_{\mathsf{max}}}(x) \equiv h_1(x; k_{\mathsf{max}})$ for linear perturbation of a ridge for no-slip $m = h^3$ (red) and intermediate-slip $m = h^2$ (blue) mobility for the corresponding most unstable wavenumber k_{max}. Full lines for alternative potential $\hat{\phi}$ and dashed lines for standard potential $\bar{\phi}$ show no significant difference. The shown eigenfunctions correspond to varicose perturbations of the ridge.

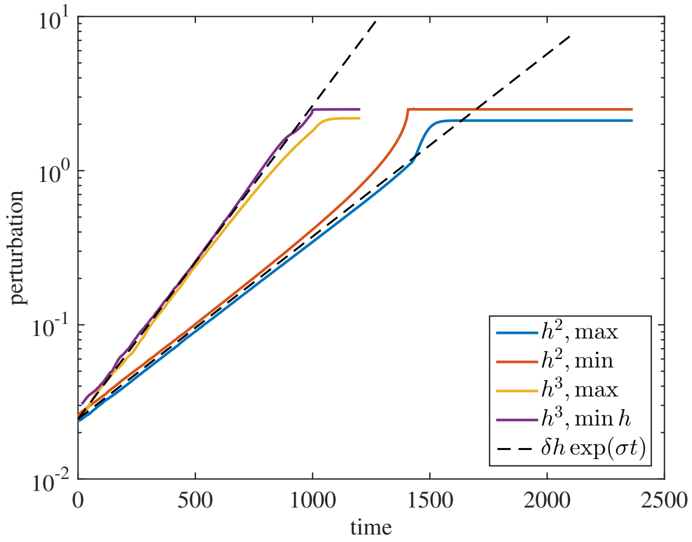

Fig. S3. Comparison of **(dashed lines)** amplification of perturbations $\delta h_1(x, k_{\mathsf{max}}) \exp(iky + \sigma(k_{\mathsf{max}})t)$ from linear stability Eq. (3) compared to **(full lines)** amplification of perturbation of the maximum on the center line $\max_y h(t,(x=0,y)) - h_{\mathsf{stat}}(x=0)$ and the minimum on the center line $\min_y h(t,(x=0,y)) - h_{\mathsf{stat}}(x=0)$ of the full numerical solution for quadratic $m = h^2$ (intermediate-slip) and cubic $m = h^3$ (no-slip) mobility indicated by different colors.

Movie S1. Energy-dissipation based model hierarchy and movies for dewetting of rims and pinch-off off ridges.
